# Supplementary figures and images for: Elucidating the regulatory role of long non-coding RNAs in drought stress response during seed germination in leaf mustard
Source: PeerJ. 2024 Jul 5;12:e17661. doi: 10.7717/peerj.17661 (PMC11229683; doi:10.7717/peerj.17661)

Dehydrin

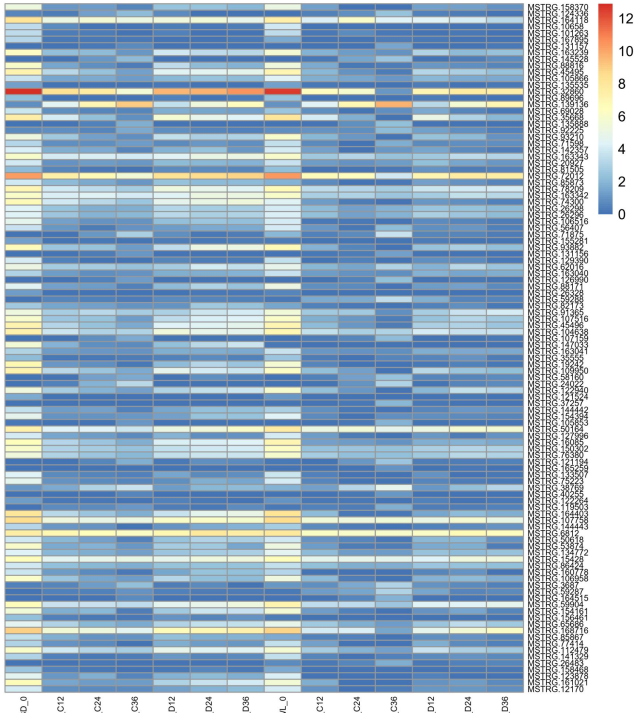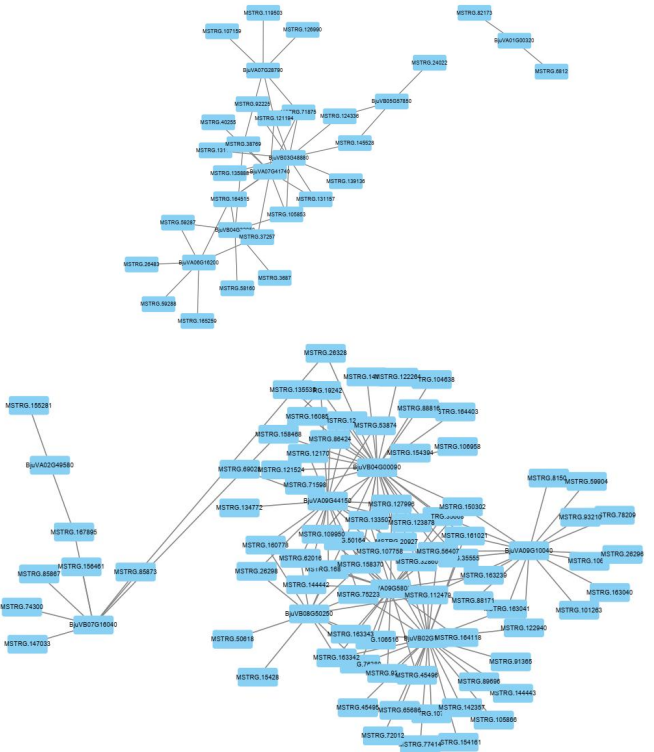

LEA\_1

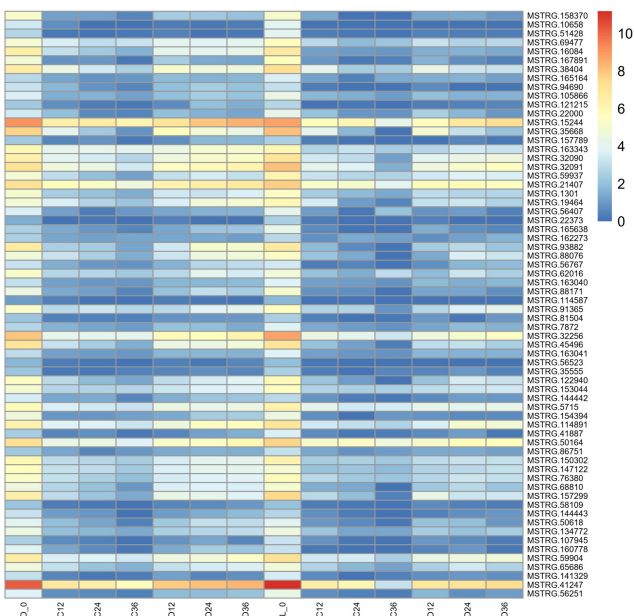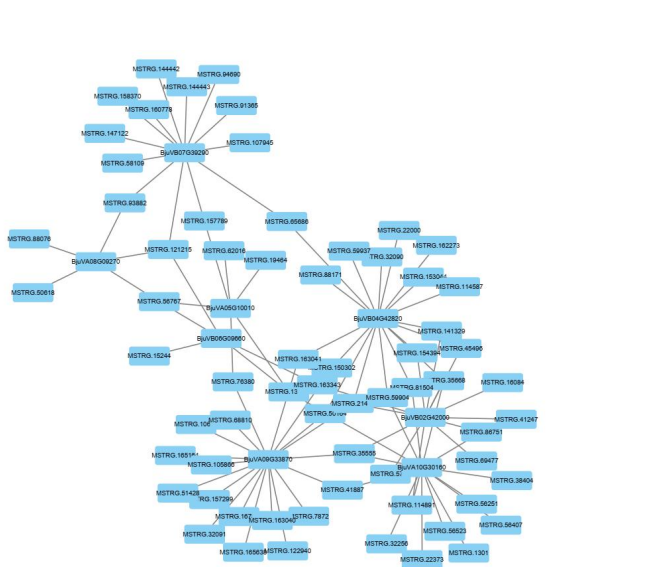

LEA\_2

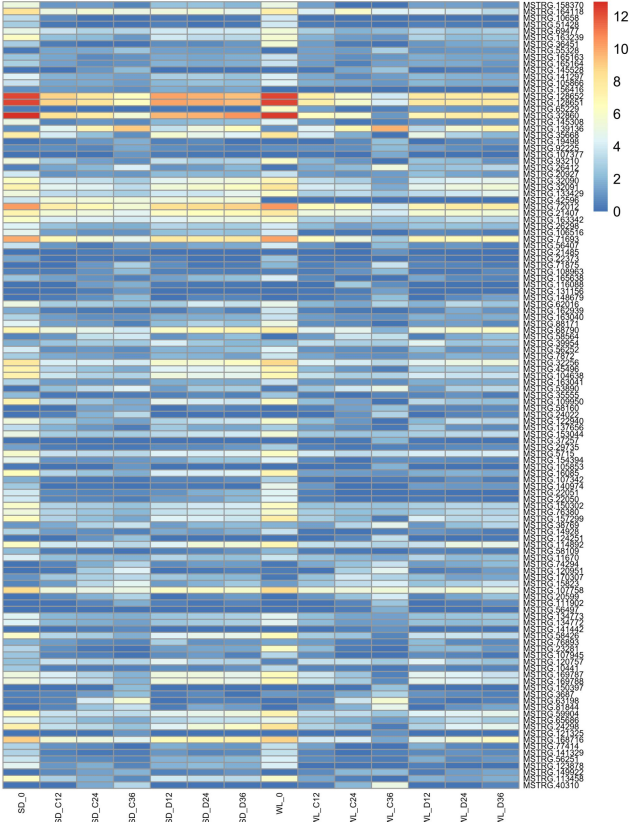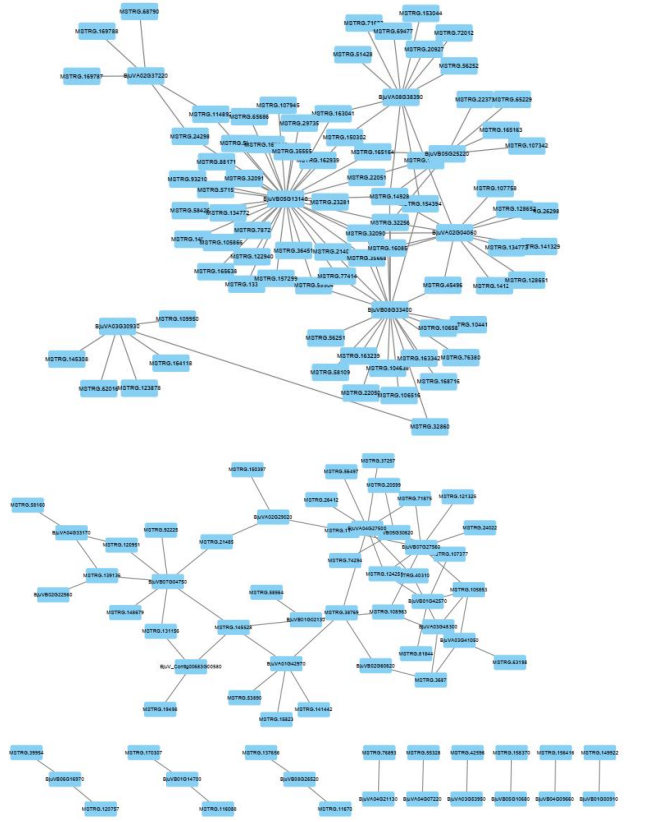

LEA\_3

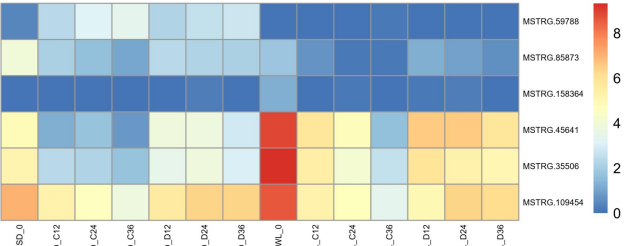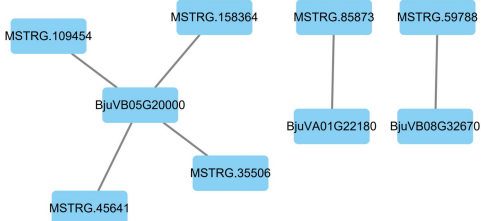

LEA\_4

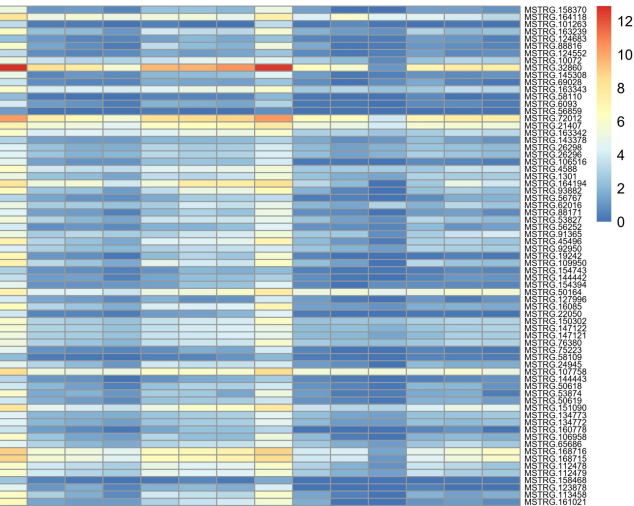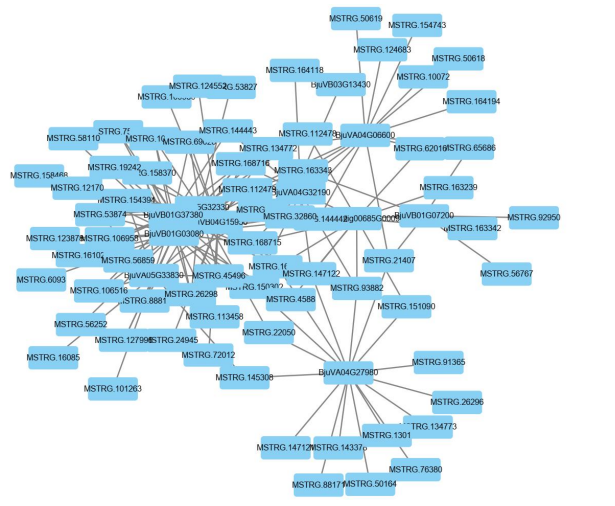

LEA\_5

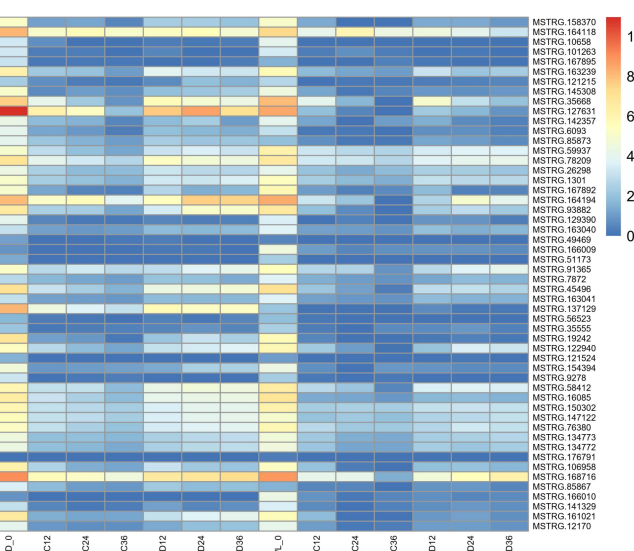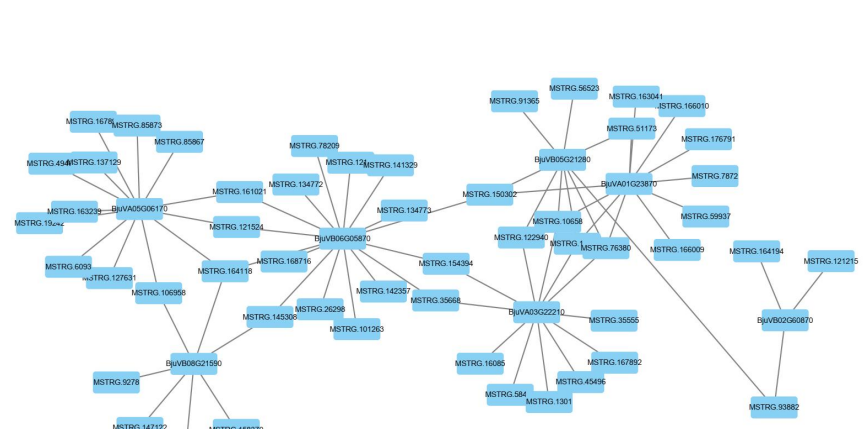

LEA\_6

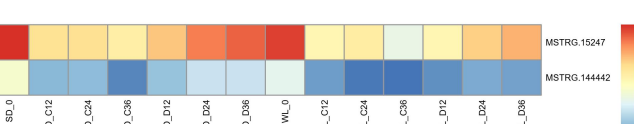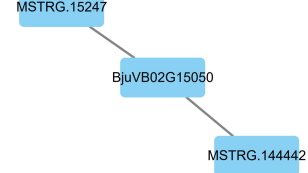

Supplement: Supplemental Information 1 [file peerj-12-17661-s001.pdf]

ATPF1G

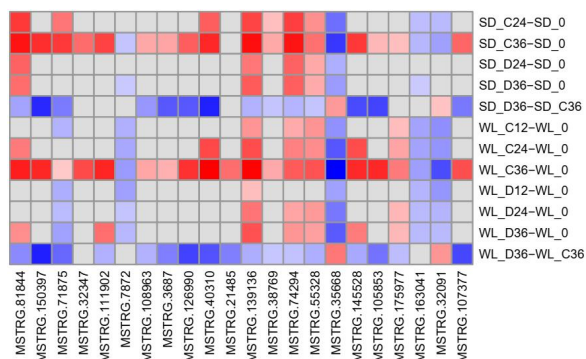

PetE

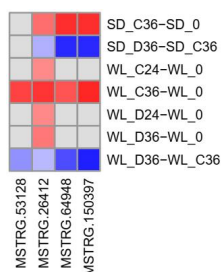

PetF

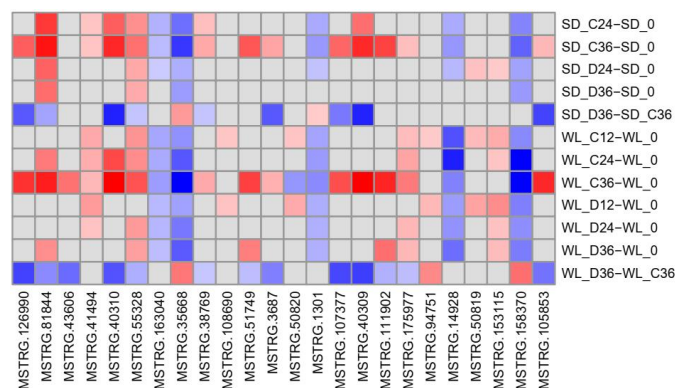

PetJ

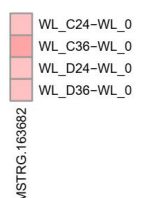

PsbO

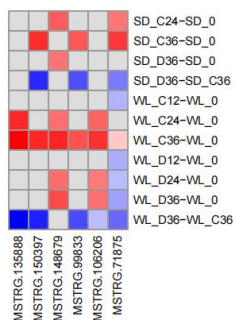

PsbP

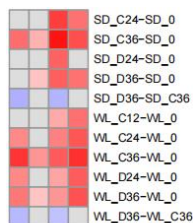

PsbY

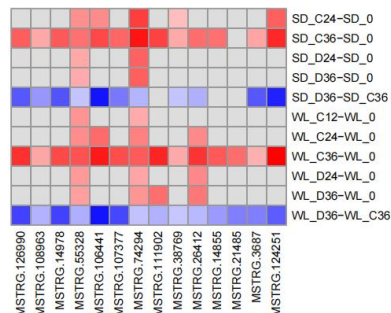

PsbR

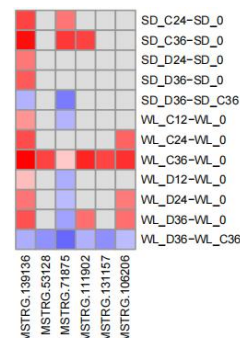

Psb28

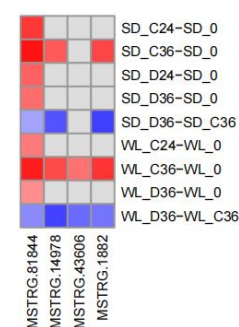

PsbS

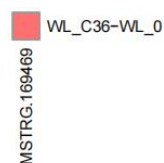

PsbQ

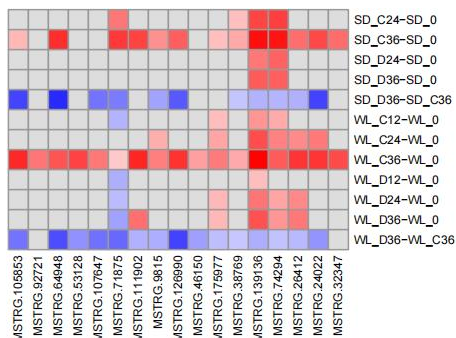

PsaC

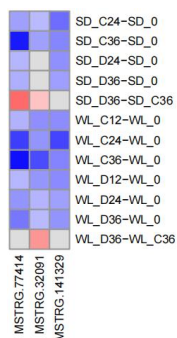

PsaE

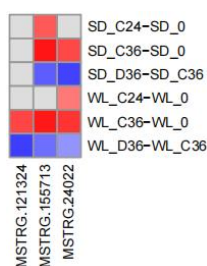

PsaF

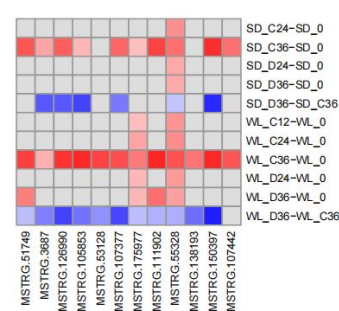

PsaH

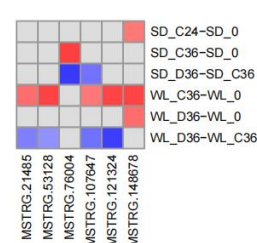

PsaK

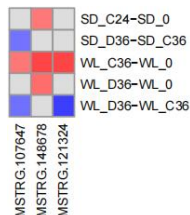

PsbW

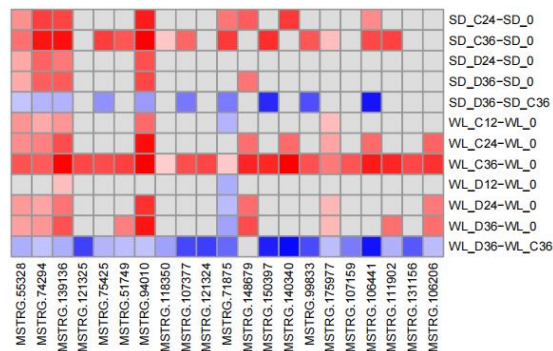

PsbG

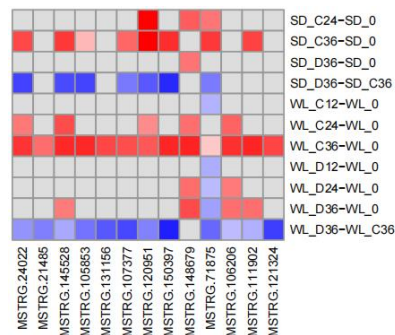

PsbO

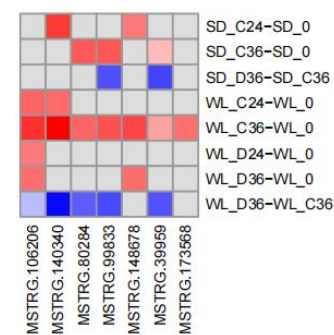

Supplement: Supplemental Information 2 [file peerj-12-17661-s002.pdf]
